# Is the Harm-to-Benefit Ratio a Key Criterion in Vaccine Approval?

**DOI:** 10.3389/fmed.2022.879120

**Published:** 2022-07-04

**Authors:** Falk Mörl, Michael Günther, Robert Rockenfeller

**Affiliations:** ^1^Forschungsgesellschaft für Angewandte Systemsicherheit und Arbeitsmedizin mbH, AG Biomechanik & Ergonomie, Erfurt, Germany; ^2^Computational Biophysics and Biorobotics, Institute for Modelling and Simulation of Biomechanical Systems, Universität Stuttgart, Stuttgart, Germany; ^3^Friedrich–Schiller–Universität, Jena, Germany; ^4^Mathematisches Institut, Universität Koblenz-Landau, Koblenz, Germany

**Keywords:** pivotal clinical trial, vaccination, SARS-CoV-2, severe adverse event, severe COVID-19 case

For the individual wellbeing or the therapy of an illness, a medical treatment should be more beneficial than harmful regarding possible adverse events of those treated. From our medical-layman point of view, until the end of 2020, we considered this part of the Hippocratic Oath, to be accepted and implemented as a rule world-wide.

Usually, concerning medical drugs, the focus of attention is on the frequency of severe adverse, i.e., potentially harmful, events (*SAE*), whose occurrences range from “very rare” (< 0.01% of treatments) to “very common” (≥ 10%) (1) . Yet, the essentials of drugs or vaccines are curing or preventing, respectively, a potentially severe illness, which would either be potentially beneficial. Apparently, it is uncommon on a broad scale to directly, quantitatively relate the frequencies of competing harmful and beneficial events; rather, (potentially rare) *SAE* and (potentially high) efficacies are communicated separately. Levels of *SAE* frequencies that might well be acceptable in administering medical drugs to cure acute illness, may not at all be acceptable for vaccines, which are administered preventively to healthy subjects. Here, a rarely occurring *SAE* may indeed spoil the preventive effect, if a severe disease progression (*SX*) of disease *X* is likewise rare. When approving the mass administration of vaccines (or even make them mandatory), one hazards the consequences of *SAE* increasing proportionally with the doses administered. The resulting balance of these potential harms and the conceived benefits, as well as the absolute number of *SAE* induced, should thus be a key criterion for the decision in favor of or against the approval of vaccines.

For the time being, in this communication, we are concerned with quantifying the basic balancing relation of harms and benefits of a vaccine introduced. To this end, let *N*_*SAE, vac*_ and *N*_*SAE, com*_ be the numbers of severe adverse events in the vaccine and comparison (often: placebo) groups, respectively, as well as *N*_*SX, vac*_ and *N*_*SX, com*_ the respective numbers of severe cases of disease *X*. Then, Δ*N*_*SAE*_ and Δ*N*_*SX*_ symbolize the additional, as compared to the occurrences in the comparison group (index “_*com*_”), number of *SAE* and the lesser number of *SX* cases, respectively, as a consequence of a given number of vaccine (index “_*vac*_”) doses. If the *harm-to-benefit ratio*


$$\begin{array}{r} hbr=\frac{N_{SAE,vac}-N_{SAE,com}}{N_{SX,com}-N_{SX,vac}}=\frac{\text{Δ}N_{SAE}}{\text{Δ}N_{SX}}=\frac{\text{Δ}{\tilde{N}}_{SAE}}{\text{Δ}{\tilde{N}}_{SX}}, \end{array}$$

with $\text{Δ}{\tilde{N}}_{SAE}=\frac{\text{Δ}N_{SAE}}{\left|N_{all}\right|}$, $\text{Δ}{\tilde{N}}_{SX}=\frac{\text{Δ}N_{SX}}{\left|N_{all}\right|}$, and the norm $|N_{all}|=\sqrt{{\left(\text{Δ}N_{SAE}\right)}^{2}+{\left(\text{Δ}N_{SX}\right)}^{2}}$, were greater than one, *SAE* due to the vaccine would more likely do severe harm to a treated person than the treated would benefit by being potentially saved from becoming severely ill due to disease *X*. Applying the same standard for *hbr* as for the frequency of *SAE* when administering medical drugs, it appears reasonable to aim at *hbr* < 0.1, i.e., at maximum one additional (short-term) *SAE* in ten prevented *SX* cases.

Our attention was attracted by the accumulating evidence of adverse events due to the world-wide SARS-CoV-2 vaccination campaign [e.g., (2)] being far more frequent than advertised initially, together with notes of the practically vanishing efficacy of the vaccines against infections with new variants of SARS-CoV-2 [e.g., (3)]. Further guided by work (4, 5) in which the *hbr* had been quantified in the field by relating mortality as a consequence of *SAE* presumably due to SARS-CoV-2 vaccination (Dutch national register “Lareb”) to COVID-19 deaths probably prevented by SARS-CoV-2 vaccination (in an Israel campaign), and being inherently inquisitive, we had a closer look at the safety and efficacy data published with the available (phase-3) papers (6–9) on pivotal clinical trials of mRNA/vector vaccines. Then simply picking up the documented counts of both severe adverse events (*N*_*SAE, vac*_, *N*_*SAE, com*_) and severe-critical COVID-19 cases (*N*_*SC*19, *vac*_, *N*_*SC*19, *com*_), in each the vaccine and comparison group of a trial, we could calculate the corresponding harm-to-benefit ratio $hbr=\frac{N_{SAE,vac}-N_{SAE,com}}{N_{SC19,com}-N_{SC19,vac}}$. Earlier, most of these trials had already been analyzed (10) with a similar approach; however, events of several severity degrees and sorts (adverse and COVID-19) had been summed within each the vaccination and the control group, therewith obscuring the individual event significances regarding risks and benefits in the trials.

One may ponder whether the grades of severity of, on the one hand, SAE due to SARS-CoV-2 vaccination and, on the other hand, “severe-critical COVID case” differ in quality. However, this is an issue to be debated on the public stage by clinical experts. Such had not been done within the pivotal clinical trial studies we have examined here. For initiation, an excessive written review of the scientific and clinical literature as well as the medical rules and guidelines currently in effect would be required indeed. In our present analysis, we imply that the use of one and the same adjective “severe” for rating both health issues in these pivotal trials indicates comparable grades of severity.

Drawing upon “severe-critical” to compare with “severe adverse” and accordingly calculating the severe-event harm-to-benefit ratio, we found that only two studies documented a ratio lower than one (Table 1), *hbr* = 0.6 or 0.9 in a particular analysis, respectively, for “Ad26.COV2.S”, as well as *hbr* = −0.4 for “Sputnik V”. The negative value for “Sputnik V” results, mathematically, from the number of *SAE* in the comparison group (receiving only the vaccine buffer composition) *exceeding* that in the vaccine group, which is a puzzling phenomenon, practically impossible for us to interpret with the information available from (9) . Figure 1 illustrates at a glance the studies' *hbr* values and their classification. It is evident that the overall short-term performance of the vaccines “BNT162b2” and “mRNA-1273”, based on the Supplementary Material published with these studies, can not be rated other than “harmful”, entirely unbalanced. “Ad26.COV2.S” performs nearly “harmful”, definitely unbalanced. A “gambling” classification is suitable, because its administration is a bet on unknown long-term harms being less likely then short-term harms already known. Astonishingly enough, the vaccine “Sputnik V” seems to perform as a “universal remedy”.

**Table 1:** Absolute counts of severe adverse events, *N*_*SAE*_, and severe cases of CoViD-19, *N*_*SC*19_, each for the vaccine (index “_*vac*_”) and the comparison (mostly: placebo) group (index “_*com*_”), as documented by the phase-3 studies (data source); the harm-to-benefit ratio *hbr* resulting from these counts, and the *Time* after full vaccination, when the count, until the end of the study, of CoViD-19 cases began; *N*_*t*_ documents the number of subjects in the vaccine group.

**Vaccine**	**Data source**	***N*_*t*_**	***N*_*SAE, vac*_**	***N*_*SAE, com*_**	***N*_*SC*19, *vac*_**	***N*_*SC*19, *com*_**	**hbr**	***Time***
		**[ ]**	**[ ]**	**[ ]**	**[ ]**	**[ ]**	**[ ]**	**[days]**
BNT162b2	(6, Figure 3 and Supplementary Tables 3, 5)	21,621	240^#^	139^#^	1	5	25^#^	14
mRNA-1273	(7, Supplementary Tables 8, 10)	15,185	234	202	0	30	1.1	14
Ad26.COV2.S	(8, Table 2 and Supplementary Table 7)	21,895	47^⋆^	21^⋆^	14	60	0.6	14
			47^⋆^	21^⋆^	5	34	0.9	28
Sputnik V	(9, Table 2)	16,427	45	(3·23=)69^†^	0	(3·20=)60^†^	−0.4	14
AZD1222	(11)	5,807	84	91^‡^	0	2	-	-

# *[Polack ei al. (6), Supplementary Table 3]: In category “serious” instead of “severe”, counts are 126 (vac) and 111 (com), thus, hbr = 3.8 . Note the one and only rating of these counts in (6, p.2608): “Few participants in either group had severe adverse events, serious adverse events, or adverse events leading to withdrawal from the trial”*.

⋆ *“AEs of interest, …” plus “AEs occurring more frequently …” plus otherwise from “SAE considered related to vaccination”*.

† *The comparison group comprises just one third the number of subjects in the vaccine group; the comparison consists of administering the vaccine buffer composition only*.

‡ *A vaccine against meningococci was administered to compare against AZD1222*.

**Figure 1:**
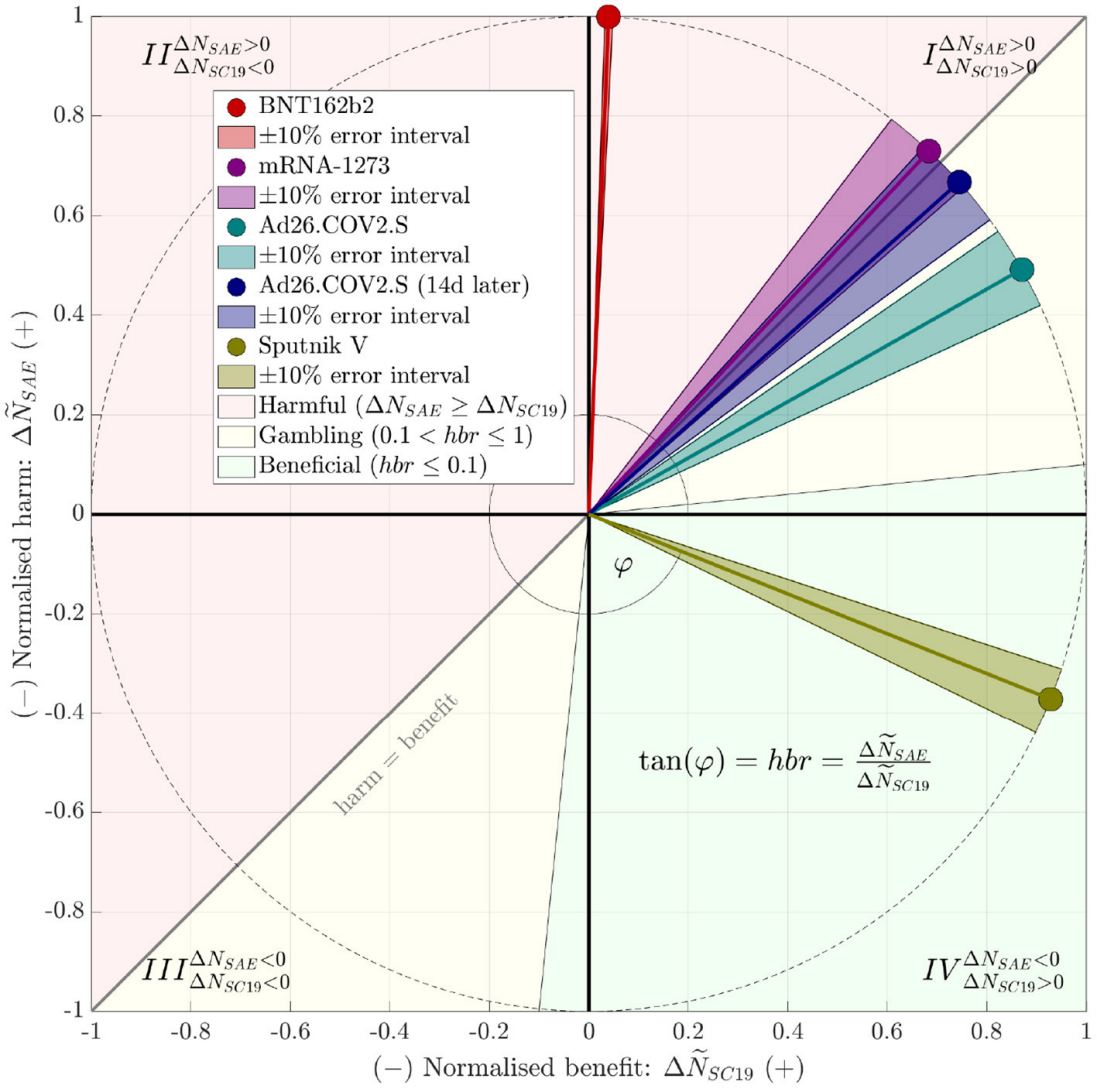
Juxtaposition, within a harm-benefit diagram, of the vaccines' observed (short-term) harm-to-benefit ratios (*hbr*) according to Table 1. To make both juxtaposed, *hbr*-contributing differences, Δ*N*_*SAE*_: = *N*_*SAE, vac*_−*N*_*SAE, com*_ (harm events) and Δ*N*_*SC*19_: = *N*_*SC*19, *com*_−*N*_*SC*19, *vac*_ (benefit events), comparable, we normalized these numbers as $\text{Δ}{\tilde{N}}_{SAE}:=\frac{\text{Δ}N_{SAE}}{\sqrt{\text{Δ}N_{SAE}^{2}+\text{Δ}N_{SC19}^{2}}}$ and $\text{Δ}{\tilde{N}}_{SC19}:=\frac{\text{Δ}N_{SC19}}{\sqrt{\text{Δ}N_{SAE}^{2}+\text{Δ}N_{SC19}^{2}}}$, respectively, for then projecting them on the unit circle. Note that the *hbr* is uniquely represented by the angle $\varphi =\text{atan2}\left(\frac{\text{Δ}N_{SAE}}{\text{Δ}N_{SC19}}\right)=\text{atan2}\left(\frac{\text{Δ}{\tilde{N}}_{SAE}}{\text{Δ}{\tilde{N}}_{SC19}}\right)$, with “atan2()” denoting the four-quadrant inverse tangent function (12). The red-shaded area above the “harm = benefit”, i.e., *hbr* = 1 line marks the harmfully unbalanced area: *hbr* > 1 indicates harm to exceed benefit in the corresponding studies. The green-shaded area marks what we would consider the “medically beneficial” (also: balanced or acceptable, respectively) area of *hbr* ≤ 0.1 (while Δ*N*_*SC*19_ > Δ*N*_*SAE*_), at least in the short term. The yellow-shaded area marks the remaining cases, which we would term “gambling” conditions, because they yield only just less short-term harm than benefit, and a vaccinated human bets on the unknown long-term harms being lower than those in the short term. Dark-shaded sectors around *hbr* pointers indicate a 10% uncertainty interval, which was constructed by disturbing numerator and denominator by 10% each and choosing the maximum and minimum resulting *hbr*. For the “Sputnik V” study (9) data, a (beneficial) negative *hbr* indicates a positive benefit alongside with a negative harm, i.e., *lesser* *SAE* in the vaccinated than in the comparison group. Note that a negative (and then harmful) *hbr* might also have occurred by doing harm (Δ*N*_*SAE*_ > 0) while being accompanied by a negative benefit (*III*nd quadrant), which had luckily not been observed in these studies.

The numbers presented in our Table 1 and Figure 1 have been widely visible since their publication. We wonder why neither a reviewer nor an approving authority seems to have noticed these jaw-dropping values of harm-to-benefit ratios. Us having missed some other vaccine-beneficial rationale would be an explanation of good nature. A crucial question has thus emerged: What scientific data and criteria other than the severe-event harm-to-benefit ratio, or rationales, have the above vaccines' approvals by authorities been exactly based on?

## Author Contributions

FM performed an initial data research and analysis, did early calculations, and wrote the initial manuscript draft. MG refined the calculation rationale and method and finally extracted all necessary numbers from the analyzed studies' data material. RR invented and drafted the graphical illustration of the calculation and results and finally generated the figure. MG and RR added passages and revised the initial draft. All authors conjointly converged iteratively by writing and discussing to the eventually submitted manuscript draft. All authors contributed to the article and approved the submitted version.

## Funding

Publication costs were covered by the Open Access Fund of the University of Koblenz-Landau.

## Conflict of Interest

The authors declare that the research was conducted in the absence of any commercial or financial relationships that could be construed as a potential conflict of interest.

## Publisher's Note

All claims expressed in this article are solely those of the authors and do not necessarily represent those of their affiliated organizations, or those of the publisher, the editors and the reviewers. Any product that may be evaluated in this article, or claim that may be made by its manufacturer, is not guaranteed or endorsed by the publisher.
